# Innovative screening and distribution strategies for addressing presbyopia

**Published:** 2026-03-12

**Authors:** Kanika Bahl, Ella Gudwin, Bradley Heslop, Pelin Munis, Abigail Steinberg, Jessica Vernon, Sumrana Yasmin

**Affiliations:** 1 CEO: Evidence Action, New York City, USA.; 2 CEO: VisionSpring, New York City, USA.; 3 Co-Founder and Co-CEO: Dot Glasses International, Nairobi, Kenya.; 4 Chief Executive Officer: RestoringVision, Miami, USA.; 5 Executive Director: Livelihood Impact Fund, Denver, USA.; 6 CEO: Maisha Meds, Nairobi, Kenya.; 7 Deputy Technical Director, Eye Health: Sightsavers, Islamabad, Pakistan.

**Multiple channels are needed to ensure everyone has access to near-vision spectacles.**

Presbyopia affects 1.8 billion people worldwide.^1^ It is easily corrected, but close to half of those affected (826 million) do not have access to the near-vision spectacles needed to correct their condition.

There is only one optometrist for every 100,000 people in eastern and western sub-Saharan Africa, and one optometrist for every 1,200,000 people in central sub-Saharan Africa.^2^ The few optometrists working there are concentrated in urban areas, with a major shortage in rural areas. As the available eye care workforce is not large enough to meet the need, other strategies are needed.

In most high-income countries, people with presbyopia have been safely self-selecting near-vision spectacles for decades, including in pharmacies, small stores, and supermarkets. However, in many low- and middle-income countries, there is little awareness of presbyopia, and too few people have access to the solution: near-vision spectacles.

Multiple strategies and channels - for both screening and distribution - are needed to ensure people have access. The more varied the channels, the greater the opportunities to provide people who have diverse needs, and live and work in different contexts, with near-vision spectacles. Here are some of the innovative screening and distribution strategies being used worldwide.

**Case scenarios: how different distribution channels can reach a wider range of people**

**Figure:**
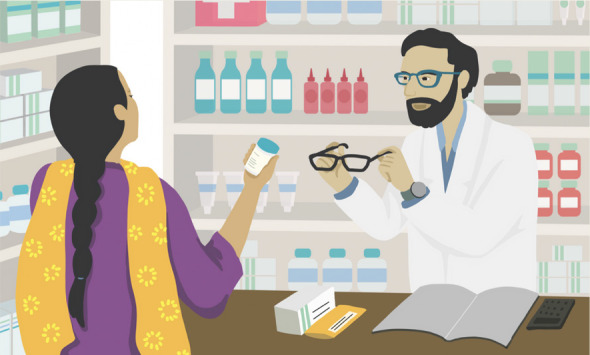
© VINA PUSPITA CC BY-NC-SA 4.0

**Sara** went to her local pharmacy to pick up her medication. While there, a pharmacist noticed she was struggling to read the instructions on the medicine bottle and prompted her to try on and select appropriate near-vision spectacles; this helped her to take her medicine safely.

**Figure:**
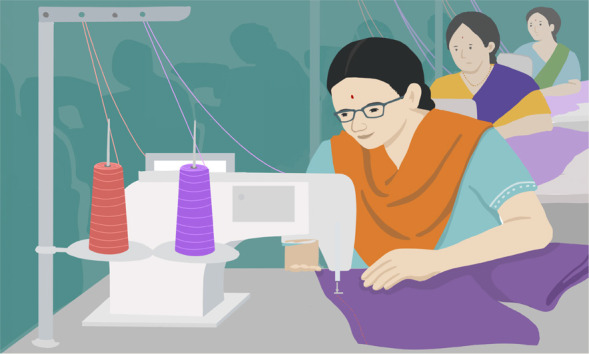
© VINA PUSPITA CC BY-NC-SA 4.0

**Nadia** works long hours in a factory making garments. She was struggling to finish as many garments per day as before, and thought she might need spectacles, but all the clinics nearby were closed by the time she left work. One day, a vision screening team came to her factory and found that she did have near-vision impairment. With near-vision spectacles, she was able to work much faster, as she could see fine details more clearly.

**Figure:**
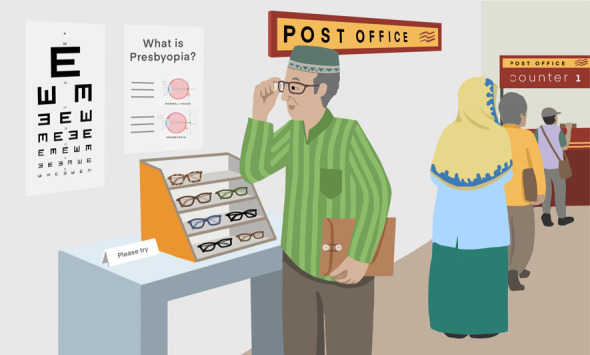
© VINA PUSPITA CC BY-NC-SA 4.0

**Deepak** had always assumed that struggling to see well up close was just a normal part of ageing, and nothing could be done to fix it. He would not have actively sought out presbyopia correction. While queuing at his local post office to send a delivery, he noticed some information about presbyopia, and a stand of spectacles to try. He tried them on and was amazed by the difference.

**Figure:**
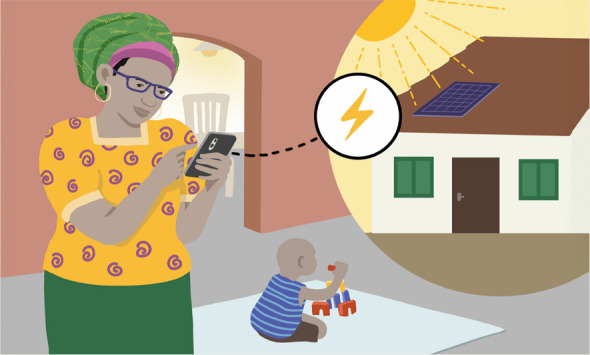
© VINA PUSPITA CC BY-NC-SA 4.0

**Mercy** spends most of her time at home, caring for her children and her elderly mother. It is difficult for her to travel into town. When Mercy's family bought a small solar panel, company staff members asked her some basic questions about her sight. After finding that Mercy had good distance vision, but struggled up close, they invited her to try near-vision spectacles. These have made many of her daily tasks much easier, including making monthly payments by phone!

*None of these distribution channels alone would have been able to reach all of these people, who have different barriers, priorities, and spend their days in different ways.*

## 1. Pharmacies

Pharmacies are a trusted point of access to care for billions of people, already providing eye health medication such as antibiotics for conjunctivitis. Maisha Meds has built technology that helps pharmacies deliver high-quality, affordable medicines while strengthening their business operations. With 5,000 pharmacies across four countries in Africa using their technology, Maisha Meds has recently partnered with suppliers of affordable spectacles, initially providing near-vision spectacles in 45 of its pharmacies in Kenya. This approach is now scaling across the four countries, aiming to provide near-vision spectacles to nearly half a million people in 2026. Maisha Meds has created pricing and incentive models for pharmacists, and it has partnered with national and local government leaders to raise presbyopia awareness in the community.

## 2. Faith-based health systems

Faith is deeply woven into the fabric of life across Africa, not only as a source of spiritual guidance, but also as a driving force for community health and development. Across the continent, Christian health associations collectively deliver between 30% and 70% of health services, depending on the country and context. In many rural areas, these health associations enable access to essential care for more than 300 million people.

Recognising their reach and trust within communities, RestoringVision partnered with the Africa Christian Health Associations Platform (ACHAP) to launch the Africa Clear Sight Partnership (ACSP), an initiative to bring vision screening and near-vision spectacles to tens of millions of people living with uncorrected presbyopia.

Through this partnership, presbyopia screening and near-vision spectacles are reaching people in need in Eswatini, Kenya, Malawi, Nigeria, Sierra Leone, and Zambia. Innovative models have emerged, such as offering access in primary and community care clinics. Eye health education has also been integrated into faith-based services: congregants learn about vision care during worship, followed by screening and spectacle distribution in tents outside the church or place of worship.

## 3. Postal workers

The Universal Postal Union (www.upu.int) reports that nearly 5 million postal workers around the globe delivered 225 billion letters and 26 billion parcels domestically in 2023, reaching both urban and remote communities. Postal workers also sell a wide range of products and are a critical service, reaching the most disadvantaged people in society: those living in the ‘last mile’ - who are not being reached by health care and eye care services.^3^

In addition to their established distribution networks and logistical experience, postal workers themselves need good near vision to do their jobs, while customers need good near vision to use their postal services. The Universal Postal Union (UPU), like the World Health Organization (WHO), is a specialised United Nations agency which works across borders with 192 member states. WHO and UPU have launched a global collaboration,^4^ starting in India, to use the postal service infrastructure to deliver presbyopia correction. This cross-sector collaboration, which is part of the WHO SPECS initiative, demonstrates how we can move beyond the health care sector to provide basic eye care.

**Figure:**
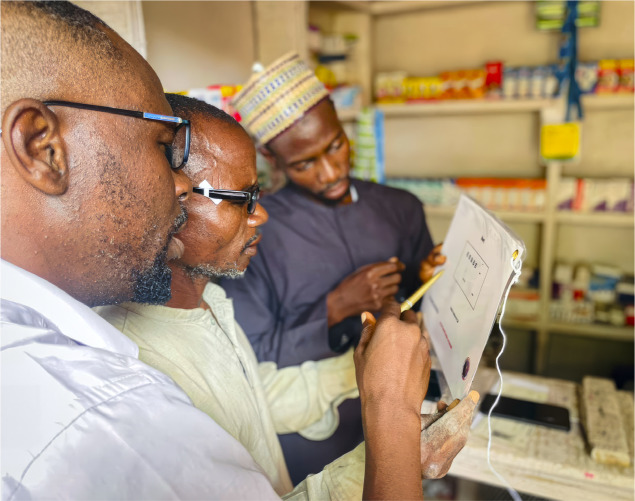
At Fadullulah Medicine Store in Sharada, pharmacy staff screen carpenter Hussaini Adam for vision problems as part of Maisha Meds’ vision programme. NIGERIA

© GRACE ADUAK/MAISHA MEDS CC BY-NC-SA 4.0

## 4. Workplaces

Due to the age of onset of symptomatic presbyopia (approximately 40 years), many people are still working and providing for their households when they begin to experience near-vision impairment.

“Due to the age of onset of symptomatic presbyopia (approximately 40 years), many people are still working and providing for their households when they begin to experience near-vision impairment.”

**Figure:**
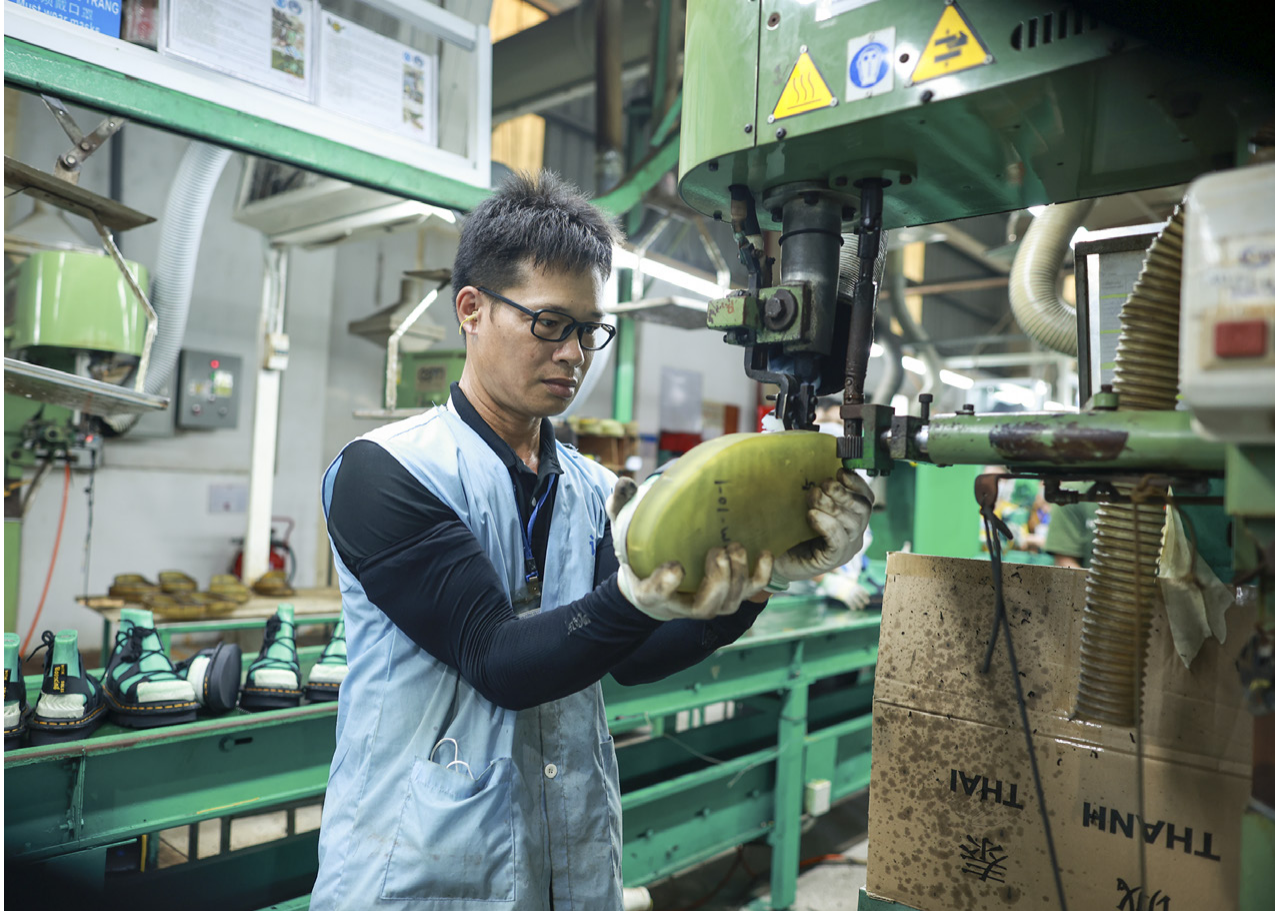
A factory worker is able to see details more clearly after receiving near-vision spectacles in his place of work. VIET NAM

© HAI NGUYEN VIA VISIONSPRING CC BY-NC-SA 4.0

Given that many health care and eye care services are provided at hospitals or clinics during working hours, working people can be disproportionately excluded from accessing these services if they are unable to leave the workplace. Finding ways to provide access to them is crucial. VisionSpring's ‘See to Earn’ initiative provides screening and spectacles - at a large scale - in workplaces, including factories, farms/agricultural estates, and via outreach into the informal sector.

## 5. Bundling services

Essential service providers, such as solar energy companies, have among the best ‘last mile’ distribution systems in low-income markets. Many already operate with microfinancing and staggered payment structures, making them well placed to reach customers who might otherwise struggle to pay for spectacles upfront.

A promising example of working with such a company to support access to presbyopia correction is the collaboration between Dot Glasses and Sun King, a leading solar energy provider. Staff members ask customers if they have a pair of near-vision spectacles; if not, the person is invited for screening and provided with near-vision spectacles, if needed. By correcting presbyopia, customers are better able to stay productive, manage repayments more efficiently, and maintain fuller participation in community life. For service providers, these improvements may translate into more consistent repayment and stronger customer relationships.

## 6. Government pension programmes

Access to eye care is essential for healthy ageing, yet millions of older adults in Peru, especially those living in poverty, lack the resources to obtain even basic vision services. Poor vision can limit independence, reduce quality of life, and increase vulnerability to injury and isolation.

To address this need, Pensión 65, the Solidarity Assistance Program of Peru's Ministry of Development and Social Inclusion, launched *Para Verte Mejor* (“To See You Better”), a nationwide initiative that offers vision screening and free near-vision spectacles to socioeconomically vulnerable older adults. The programme is implemented in collaboration with Management Sciences for Health (MSH)-Perú and RestoringVision. To date, the programme has reached 414,000 people across Peru in an efficient and sustainable way.

## 7. Outreach vision camps

Sightsavers partners with ministries of health and local organisations to hold outreach vision camps that identify people with vision needs, especially in marginalised communities. To ensure as many people as possible feel comfortable attending the screening camps, they are set up in locally accepted spaces within walking distance of people's homes, such as schools. Attendees undergo near and distance vision screening and an eye examination. People who need surgery are referred, and those who need spectacles or an alteration to their current prescription are seen by an optometrist or refractionist, who prescribes spectacles.

By bringing services closer to communities, these camps have improved access to, and acceptance of, eye health services and spectacles in remote and rural communities, particularly for women, girls, older people, and people with disabilities.

“To ensure as many people as possible feel comfortable attending the screening camps, they are set up in locally accepted spaces within walking distance of people's homes, such as schools.”

## 8. Making the most of existing programmes outside of eye care

Evidence Action runs a water, sanitation, and hygiene (WASH) programme in Mbale district in Uganda, which reaches 10% of Uganda's population. In August 2025, they launched a two-armed near-vision spectacle distribution project that will run until early 2026. In the second arm, Evidence Action personnel, who already promote safe water practices and chlorination uptake, now host near-vision screening and spectacle distribution events in the communities they serve. Staff members work with local radio, community health workers, local leaders, and key community members to inform people about the event and ensure as many people as possible attend.

**Figure:**
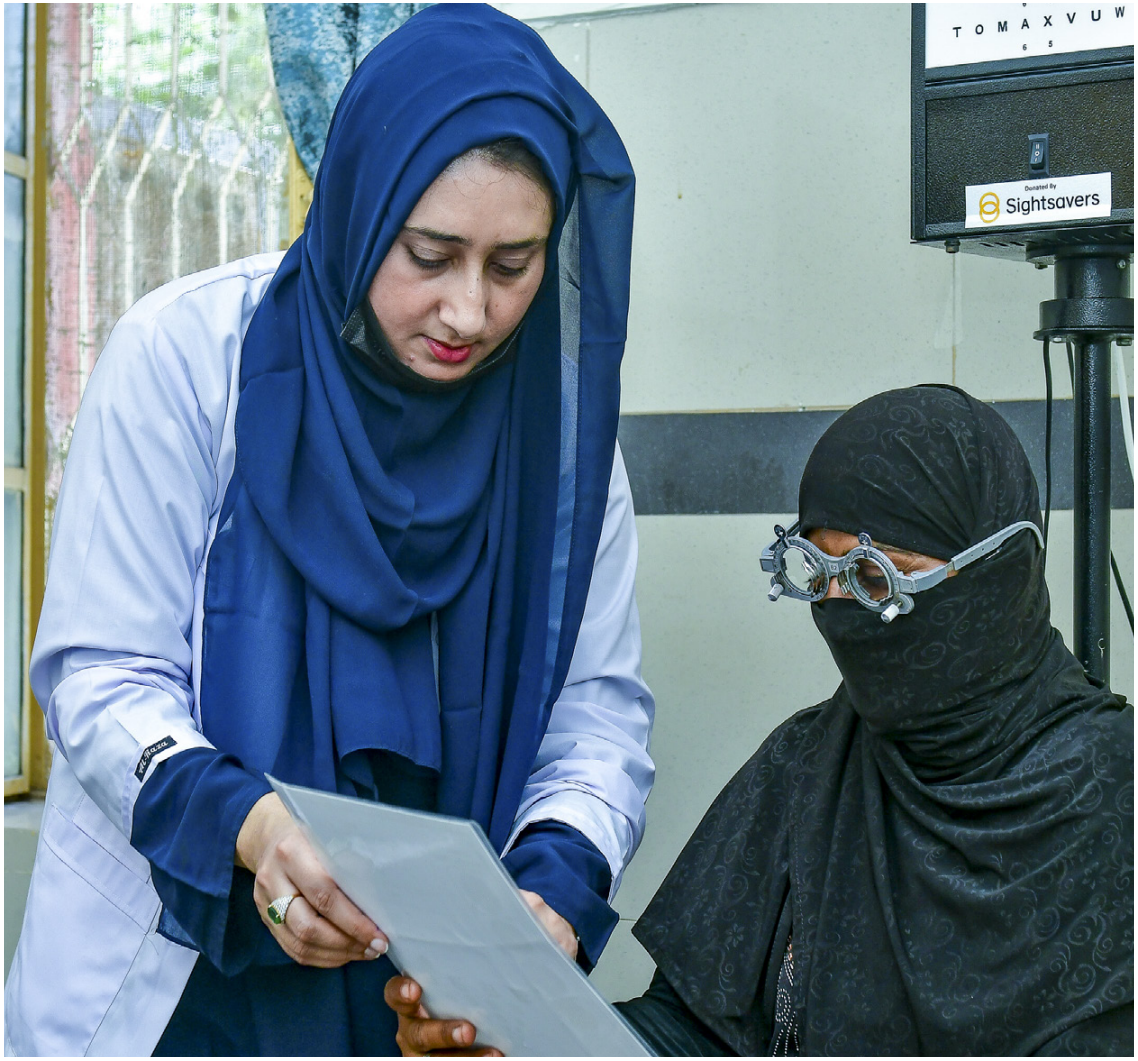
Outreach camps improve equitable access to near-vision services. PAKISTAN

© SIGHTSAVERS/JAMSHYD MASUD CC BY-NC-SA 4.0

**Further reading**

To read more insights into how other organisations are distributing near-vision spectacles, visit bit.ly/3qTyBzt.
